# Could Stoma Be Avoided after Laparoscopic Low Anterior Resection for Rectal Cancer? Experience with Transanal Tube in 195 Cases

**DOI:** 10.3390/jcm11092632

**Published:** 2022-05-07

**Authors:** Antonio Sciuto, Roberto Peltrini, Federica Andreoli, Andrea Gianmario Di Santo Albini, Maria Michela Di Nuzzo, Nello Pirozzi, Marcello Filotico, Federica Lauria, Giuseppe Boccia, Michele D’Ambra, Ruggero Lionetti, Carlo De Werra, Felice Pirozzi, Francesco Corcione

**Affiliations:** 1 Department of General Surgery, Santa Maria delle Grazie Hospital, 80078 Pozzuoli, Italy; felice.pirozzi@aslnapoli2nord.it; 2 Department of Electrical Engineering and Information Technology, University of Naples Federico II, 80125 Naples, Italy; 3 Department of Public Health, University of Naples Federico II, 80131 Naples, Italy; roberto.peltrini@gmail.com (R.P.); disanto.albini@gmail.com (A.G.D.S.A.); mariamichela.dinuzzo@outlook.it (M.M.D.N.); nellopirox@gmail.com (N.P.); marcello.filotico@unina.it (M.F.); federica.lauria91@gmail.com (F.L.); giuseppe.boccia.29@gmail.com (G.B.); michele.dambra@unina.it (M.D.); ruggero.lionetti@unina.it (R.L.); dewerra@unina.it (C.D.W.); francesco.corcione@unina.it (F.C.); 4 Department of Minimally Invasive Surgery, Cristo Re Hospital, 00167 Rome, Italy; andreoli.fede@gmail.com

**Keywords:** transanal tube, low anterior resection, anastomotic leakage, defunctioning stoma, transanal stent, colorectal surgery, colorectal cancer, laparoscopy, postoperative outcomes

## Abstract

Anastomotic leakage is the most-feared complication of rectal surgery. Transanal devices have been suggested for anastomotic protection as an alternative to defunctioning stoma, although evidence is conflicting, and no single device is widely used in clinical practice. The aim of this paper is to investigate the safety and efficacy of a transanal tube for the prevention of leakage following laparoscopic rectal cancer resection. A transanal tube was used in the cases of total mesorectal excision with low colorectal or coloanal anastomosis, undamaged doughnuts, and negative intraoperative air-leak test. The transanal tube was kept in place until the seventh postoperative day. A total of 195 consecutive patients were retrieved from a prospective surgical database and included in the study. Of these, 71.8% received preoperative chemoradiotherapy. The perioperative mortality rate was 1.0%. Anastomotic leakage occurred in 19 patients, accounting for an incidence rate of 9.7%. Among these, 13 patients underwent re-laparoscopy and ileostomy, while 6 patients were managed conservatively. Overall, the stoma rate was 6.7%. The use of a transanal tube may be a suitable strategy for anastomotic protection following restorative rectal cancer resection. This approach could avoid the burden of a stoma in selected patients with low anastomoses.

## 1. Introduction

Anastomotic leakage (AL) is the most-feared complication following rectal surgery, especially after lower anastomoses [1,2]. It is associated with high postoperative morbidity and mortality as well as poor long-term survival and functional outcomes [3,4,5,6]. Several factors may contribute to leakage, also including intraluminal rectal pressure and fecal load. With this background, a temporary protective stoma for fecal diversion has been suggested for low rectal resections [7,8,9,10,11]. However, significant morbidity and costs may result from a stoma and its subsequent reversal [12,13,14]. Therefore, alternative procedures to reduce or prevent AL and concomitant sequelae have been suggested. There are several successful reports of different intraluminal devices, including a transanal tube (TT) or transanastomotic stent, which may protect anastomosis against leakage [15,16,17,18,19,20,21,22,23,24,25,26]. However, no single device is widely used in clinical practice [27]. Evidence on the efficacy of a TT in reducing the leakage rate after low rectal resection is still conflicting. Furthermore, some RCTs also included patients who received a diverting stoma in the TT group [28,29]. Therefore, the aim of this paper is to investigate the safety and efficacy of the exclusive application of a TT for the prevention of AL following laparoscopic low anterior resection for rectal cancer.

## 2. Materials and Methods

**Figure 1:**
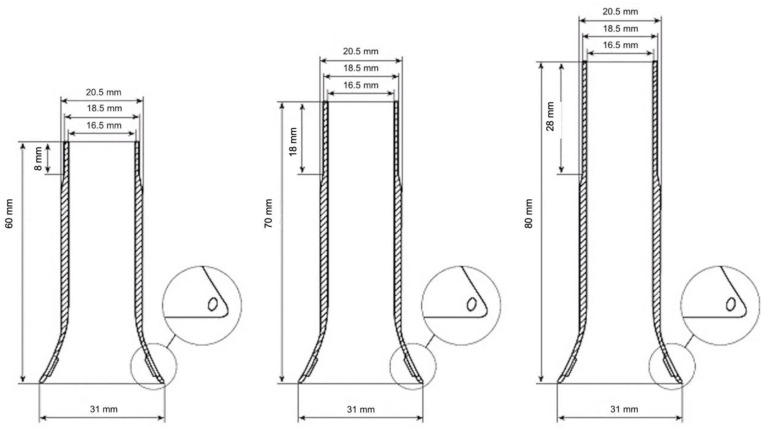
Technical features of the No Coil tube.

A retrospective review of a prospectively maintained single-center database was performed. All consecutive patients who received a TT (No Coil, Sapimed, Alessandria, Italy) for anastomotic protection were included. The data were collected from hospital charts and operative reports. The TT was used in the cases of total mesorectal excision (TME) with low colorectal or coloanal anastomosis, undamaged doughnuts, and negative intraoperative air-leak test. The device is a soft silicone tube, slightly cone shaped, with variable length (Figure 1).

After colonoscopy and histologically proven mid–low rectal tumor, preoperative clinical staging was accomplished by a CT scan of the chest, abdomen, and pelvis, and loco-regional imaging evaluation, including pelvic MRI scan and transrectal ultrasound when necessary. Postoperative mortality, hospital stay, 30-day leakage-related morbidity, and rates of reoperation and stoma were recorded. Descriptive statistics were reported as number and percentages for categorical variables and mean ± SD for continuous variables.

The primary endpoint was the postoperative AL rate within 30 days. Clinical anastomotic leakage was considered present when a patient developed (1) peritonitis and related abnormalities: pelvic or perineal pain or tenderness, tachycardia, fever, and increased white blood cell count; (2) gas, fecal, or purulent discharge from the pelvic drain, drain tract, or anus; (3) pelvic abscess or fluid collection; (4) rectovaginal fistula. The severity of AL was graded according to the impact on clinical management (as reported by the International Study Group of Rectal Cancer) [30], as follows: grade A, not requiring active therapeutic intervention (also classified by several authors as “radiologic leakage”); grade B, requiring active therapeutic intervention, but manageable without reoperation; grade C, requiring operative reintervention. Radiologically demonstrated leakage without clinical symptoms (grade A) was not included in the analysis. This study was conducted according to the STROBE guidelines [31].

### Surgical Technique

**Figure 2:**
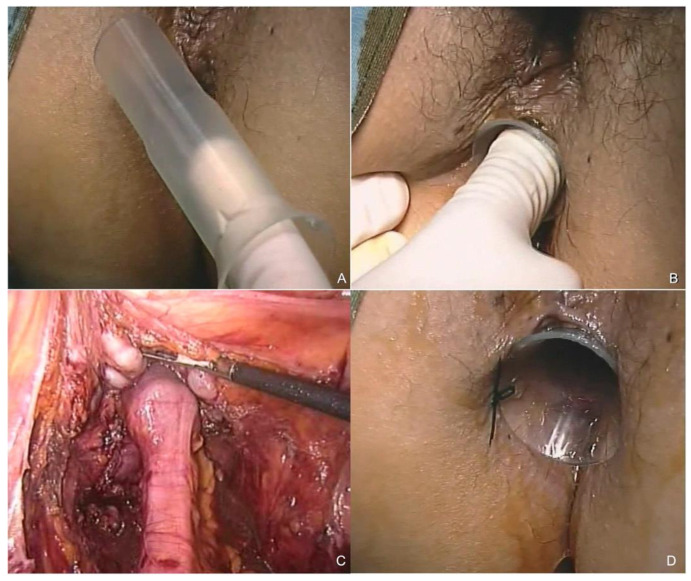
After the anastomosis has been performed, the lubricated tube (**A**) is inserted into the anal canal and placed to coat the anastomotic area, until the tube’s tongues contact the perineal skin (**B**). The proper position of the tube is checked by means of concomitant laparoscopic view (**C**). Final view after securing the tube to the perianal skin by two silk stitches (**D**).

Bowel preparation included a liquid diet and phosphate enemas on the day prior to surgery. The procedure began with coloepiploic detachment, identification of the inferior mesenteric vein at the lower border of the pancreas, and then medial-to-lateral dissection between the Toldt and Gerota fasciae. A full splenic flexure mobilization as well as a high ligation of the inferior mesenteric artery were always performed. TME was carried out. Rectal dissection began posteriorly at the pelvic brim and was continued in the avascular plane as far distally as possible. TME proceeded by scoring the peritoneal leaves on each side of the rectum to the lateral ligaments, which were divided. Then, anterior dissection was accomplished. Finally, the division of the rectosacral ligament allowed complete rectal mobilization all the way to the pelvic floor. At this level, the rectum was transected using a 45 mm linear stapler and, after specimen extraction through a suprapubic incision, a colorectal anastomosis was performed using the double stapling technique. Otherwise, after exposing the anal canal with a retractor, an intersphincteric dissection was performed. The rectum was extracted through the anus and a hand-sewn coloanal anastomosis was fashioned with interrupted stitches. A pelvic drain was placed close to the anastomosis in all patients. After the anastomosis was complete, a No Coil tube was gently inserted into the anus and placed to coat the anastomotic site until the tube’s tongues contacted the perineal skin. The proper position of the tube was checked by concomitant laparoscopic view. Finally, the tube was secured to the perineal skin by two silk stitches (Figure 2). The TT was kept in place until the 7th postoperative day. The abdominal drain was removed the following day if no signs of leakage occurred. When a postoperative complication required reoperation, a minimally invasive approach was preferred [32].

## 3. Results

Between March 2004 and December 2013, 386 patients underwent elective laparoscopic anterior resection for primary rectal cancer. A total of 108 (55.4%) males and 87 (44.6%) females with a mean age of 58.2 ± 9.3 years received a TT tube and were included in the study (Table 1).

In 102 cases (52.3%), the tumor was in the medium rectum (10 to 5 cm from the anal verge), and in 93 cases (47.7%), it affected the low rectum (<5 cm from the anal verge). Neoadjuvant chemoradiotherapy was carried out in 140 (71.8%) patients who underwent surgery 6 to 8 weeks after the end of the treatment. A double stapling end-to-end anastomosis was performed in 182 cases (93.3%), while 13 patients (6.7%) had a hand-sewn coloanal anastomosis after intersphincteric resection.

Two patients died because of pulmonary embolism and myocardial infarction, respectively, accounting for a perioperative mortality rate of 1.0%. Mean hospital stay was 12 ± 2.4 days. In two patients, the stent did not remain in situ for the planned 7 days, because it fell out on the 3rd and the 5th postoperative day, respectively. The former patient had an uneventful subsequent course, while the latter developed AL on the following day and underwent reoperation with fashioning of ileostomy.

**Figure 3:**
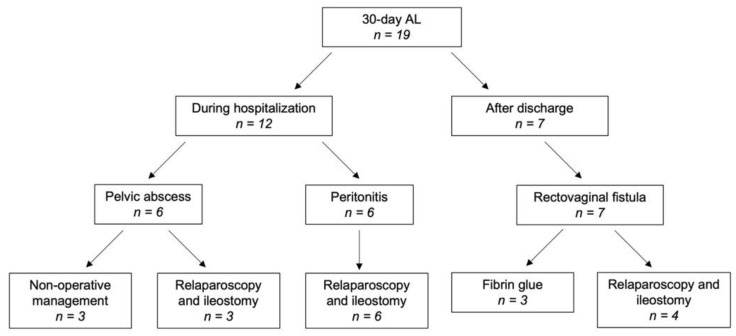
Anastomotic leakage after laparoscopic TME and transanal tube: clinical presentation and management.

AL occurred in 19 of the 195 patients, accounting for an incidence rate of 9.7%. The features of the patients affected by leakage are reported in Table 2. Nine patients (AL Grade C) underwent relaparoscopy with peritoneal lavage, drain placement, and fashioning of a defunctioning ileostomy. All of them had their stoma reversed between 38 and 47 days after surgery. Two of these patients subsequently developed anastomotic stricture that was successfully treated by endoscopic dilation. Non-operative management with total parenteral nutrition and antibiotics was adopted for three patients (AL grade B). Healing was observed after 14, 16, and 21 days, respectively. Seven patients were readmitted because of rectovaginal fistula between the 20th and the 28th postoperative day. Three cases of low-output fistulas were successfully treated by repeated fibrin glue applications, while the remaining four required a defunctioning ileostomy that was reversed 60, 75, 85, and 103 days after surgery, respectively (Figure 3). Overall, the stoma rate was 6.7%. Minor complications related to the TT placement included transient minor incontinence and perianal dermatitis.

## 4. Discussion

Although minimally invasive surgery has improved postoperative outcomes [33,34], AL remains the most significant complication after sphincter-saving surgery for rectal cancer, with an incidence that has been reported as ranging from 1% to 24% [35,36]. Leakage is the major cause of postoperative mortality and morbidity and usually requires reoperation. The mortality rate associated with AL varies from 2.1% to 22% [29]. Furthermore, impaired long-term anorectal function, greater prevalence of local recurrence, and poorer long-term survival have been clearly demonstrated as consequences of AL [3,4,5,6].

The etiology of AL is multifactorial and both systemic and local factors may play a causative role [1]. The level of the anastomosis above the anal verge appears to be the main determinant of leakage in rectal surgery. Other significant risk factors include advanced age, male gender, obesity, malnutrition, preoperative weight loss, diabetes, cardiovascular disease, steroid use, perioperative blood transfusion, mechanical bowel preparation, tumor stage and grading, and neoadjuvant therapy [27,36,37,38,39,40,41]. AL has also been shown to be related to the skills and experience as well as the specialization of the surgeon performing the operation.

Certainly, some technical prerequisites for constructing colorectal anastomoses, such as an adequate blood flow and the absence of tension, are essential to avoid leakage. Many different techniques of anastomosis have been described in search of the safest method. Furthermore, significant advances have been made in understanding the perioperative factors that predispose patients to AL. However, little is known about the optimal procedure to prevent this complication [42].

A defunctioning stoma, a conventional fecal diversion method, is commonly used when anastomotic leakage is a concern. It has been argued that the stoma is an effective procedure to prevent AL. Theoretically, a diverting stoma protects the anastomotic site from the fecal load, thereby preventing further leakage of stools into the peritoneal cavity when anastomotic dehiscence occurs. Thus, fecal diversion mitigates the catastrophic septic complications of AL and reduces both reoperation and mortality rates [7,8,9,10,11]. However, controversy surrounds the question of whether all patients with a low anastomosis should have a temporary stoma, or whether a selective approach is optimal [43]. Shielding the anastomosis from contact with stools might also reduce the incidence of AL. In several experimental studies [15,16,17,18,19], Ravo demonstrated that fecal load could impair the healing of a colonic anastomosis. Feces may be responsible for a peri-anastomotic inflammatory process which, in turn, may compromise anabolic collagen deposition and so complete healing of the colonic stumps, thus increasing the likelihood of AL.

However, a stoma requires the patient to undergo a second surgical trauma. Furthermore, the construction of a stoma as well as its subsequent reversal are associated with failure, complications, and even mortality [12]. Some studies have reported considerable stoma-related complications, occurring in up to 33% of cases. They include dermatitis, skin infection, bleeding, high stoma output, stricture or prolapse of the stoma, enterocutaneous fistula, and parastomal hernia. Necrosis of the stoma, sub-occlusive crises before the stoma closure, and at least patient discomfort have also been described [12,13,14]. Although stoma closure is often thought to be a simple procedure, it requires hospital readmission with increased costs and may have a significant impact on the patient. Several studies have described high morbidity after stoma reversal, including anastomotic dehiscence, small bowel obstruction, incisional hernias, wound dehiscence, anal stricture, and fecal incontinence [14,44]. Finally, several temporary stomas tend to be left in situ for much longer than initially anticipated, sometimes even for life [12,45].

With this background, an alternative procedure with at least equivalent effectiveness and lower morbidity would be beneficial. Different types of intraluminal bypass have been described for fecal diversion [27]. They were tested in animal experiments with successful results. In addition, several preliminary clinical studies have demonstrated that intraluminal devices were a viable alternative to a temporary stoma, decreasing the morbidity, mortality, psychological problems, and economic costs associated with multiple-stage procedures [19,20,21,22,23,24].

Other authors have emphasized the role of intraluminal pressure above the anus in impairing anastomotic healing [20,22,23,46]. In the first postoperative days, an increased intraluminal rectal pressure because of a tightly closed anal sphincter may theoretically result in fecal extrusion through an anastomosis not yet consolidated. This could be one of several potential factors in the pathogenesis of AL. Transanal decompression devices were thus designed [20,46]. A TT may prevent leakage by keeping the anal sphincter open, thus decreasing the intraluminal pressure and therefore the pressure on the anastomosis. Hence, the potential role of a TT is supposed to be beneficial for both the reduction of endoluminal pressure and fecal diversion, resulting in a protective effect on anastomotic healing. In a preliminary study evaluating the role of endoluminal pressure in AL, Montemurro et al. observed a reduction in the incidence of leakage using a TT [20]. Moreover, in a retrospective series of 184 patients [46], the authors reported a clinical AL rate of 4.8%, with no leakage-related mortality and a stoma rate of 2.7%. Thus, they concluded that the use of a TT could be a valid option for anastomotic protection after cancer proctectomy.

Some clinical randomized trials have been designed to investigate the role of transanal tubes in the prevention of leakage. In 2011, Xiao et al. [23] published a study of 398 patients who were randomized to TT (*n* = 200) or not (*n* = 198) after low anterior resection for rectal carcinoma. The overall rate of symptomatic AL was 6.78%. The incidence was significantly lower in patients with a TT (4.0% vs. 9.6%, *p* = 0.026). Mortality was nil in the study population. Regarding the double-stapled technique subgroup, patients with a tube had a significantly lower incidence of symptomatic leakage (3.7% vs. 9.3%, *p* = 0.028) and need for urgent abdominal reoperation for leakage (28.6% vs. 82.4%, *p* = 0.021). Quicker resumption of gastrointestinal motility, lower rectal resting pressure, and shorter length of hospital stay were associated with the use of a TT. In 2006, Bulow et al. [24] published a trial including 194 patients who underwent anterior resection for mobile tumor < 15 cm above the anal verge. The use of a protective ileostomy was left to the discretion of the operating surgeon. At the end of surgery, the patients were randomized into two groups with and without a transanal stent. An interim analysis was performed that showed more leakages in the stent (17%) group than in the control group (8%), although it was not statistically significant (*p* = 0.09). The study was prematurely discontinued for ethical reasons because of this trend. In 2003, a randomized trial by Amin et al. [22] compared the proximal defunctioning loop stoma with transanal stent in patients undergoing TME for rectal cancer. In total, 42 of 118 patients were not randomized because of high-dose preoperative radiotherapy, concern about the anastomosis, or obstructing tumors; 76 patients were randomized: 41 to stent and 35 to stoma. No significant difference in AL rate was demonstrated between the two groups. However, the study suffers from unclear eligibility criteria and randomization strategy, and no control group with patients without stoma or stent was described.

In 2015, Hidaka et al. [25] published a retrospective series on 205 patients who underwent laparoscopic low anterior resection without diverting stoma or preoperative chemoradiation. The leakage rate was significantly lower (*p* < 0.001) in the TT group (4.2%) than in the patients operated on without a TT (13.8%). Moreover, the reoperation rate for symptomatic AL was 73.3% in the latter group, while none of the patients who received a TT needed reoperation for leakage (*p* < 0.05). Similarly, in a retrospective study [26] on 176 patients without diverting stoma, AL was significantly associated with the non-use of a TT (OR 11.1, 95% CI 1.04–118; *p* = 0.04). However, the authors advised that complications associated with a TT, including bowel perforation, should be considered.

In the last few years, two multicenter RCTs were conducted. In a cohort of 560 patients who did not receive neoadjuvant therapy, Zhao et al. concluded that TT may not confer any benefit for AL prevention in patients undergoing laparoscopic low anterior resection for rectal cancer [28]. Likewise, in an RCT involving 157 patients from six Japanese hospitals, TT had no significant benefit in terms of the prevention of AL, despite diverting stoma also being performed in about half of the study population [29].

The clinical AL rate of 9.7% reported in our study compares favorably with the results of other investigators [47]. Sixty-three percent of patients with AL underwent reoperation. However, none of them required open surgical revision. Mild peritoneal contamination and good status of the anastomoses allowed the performance of peritoneal lavage and ileostomy in all cases, without taking down the anastomosis. Moreover, differently from other studies, patients undergoing neoadjuvant treatment were included in the analysis. In our series, the stoma rate was 6.7%, so the absence of a diverting stoma in 93.3% of patients could be considered satisfactory. Using this alternative strategy for anastomotic protection may strengthen the reduced invasiveness of the laparoscopic approach for low anterior resections. However, this is a retrospective report that does not compare the efficacy of transanal stent to that of a diverting stoma.

Venous thromboembolism and cardiovascular disease must be considered in patients with benign or malignant tumors who undergo surgery or neoadjuvant therapy [48,49]. In the present series, no mortality from AL occurred, while two deaths in hospitalized patients were due to medical complications.

Early discharge after elective colorectal surgery is currently considered feasible and safe in the setting of enhanced recovery pathways [50,51]. The mean hospital stay was 12 days in the present study. It should be noted that an enhanced recovery pathway was not adopted in this cohort of patients and that the TT was removed on the seventh day after surgery. The optimal duration for which a TT should be kept in place is unclear, although most authors suggest 5 to 7 days after the operation [22,23,25,26,29,46]. However, earlier removal (3 to 4 days) has also been reported [24,28]. Moreover, among patients who suffered from leakage following a TT in our series, 36.8% developed clinical manifestations after discharge and were readmitted for rectovaginal fistula. The rate (3.5%) of rectovaginal fistula appears to be higher than that reported by other studies [52]. This might prompt careful consideration of TT use in female patients and deserves further research. Nevertheless, it should be noted that AL often causes rectovaginal fistula when an intrapelvic abscess penetrates the posterior vaginal wall. Patient selection for TT use is challenging and additional evidence on the topic is warranted. Such studies could allow the identification of some categories of patients that may benefit more from the use of a stent, thus reserving a selective approach for defunctioning stoma. Based on the present series, younger male patients with few or no associated risk factors for AL might be the best candidates for the use of TT.

Limitations to our study include the retrospective nature leading to possible selection bias and the lack of a comparative group.

## 5. Conclusions

The use of a transanal tube is a suitable strategy for anastomotic protection following restorative rectal cancer resection. This approach could avoid the burden of a stoma in selected patients with low anastomoses. Further evidence from randomized studies is needed to clarify its safety and efficacy, taking into account preoperative chemoradiation, the absence of a diverting stoma and an adequate follow-up, as well as the integration into enhanced recovery pathways.

## Figures and Tables

**Table 1:** Perioperative characteristics of the 195 patients who had laparoscopic TME and transanal tube for rectal cancer.

	All Patients (*n* = 195)
Gender	
*Male*	108 (55.4%)
*Female*	87 (44.6%)
Age (years)	58.2 ± 9.3
BMI (Kg/m^2^)	22.3 ± 4.1
Tumor location	
*Medium rectum*	102 (52.3%)
*Low rectum*	93 (47.7%)
Pathologic tumor stage	
*T1*	29 (14.9%)
*T2*	73 (37.4%)
*T3*	85 (43.6%)
*T4*	8 (4.1%)
Neoadjuvant therapy	140 (71.8%)
Type of anastomosis	
*Double stapling technique*	182 (93.3%)
*Coloanal hand-sewn*	13 (6.7%)
Anastomotic leakage	19 (9.7%)
Displaced transanal tube	2 (1%)
Hospital stay (days)	12 ± 2.4
Mortality	2 (1%)

**Table 2:** Characteristics of the patients affected by anastomotic leakage after laparoscopic TME and transanal tube.

	Patients with Leakage (*n* = 19)
Gender	
*Male*	9 (47.3%)
*Female*	10 (52.6%)
Age (years)	62.2 ± 8.1
BMI (Kg/m^2^)	23.0 ± 1.9
Pathologic tumor stage	
*T1*	7 (36.8%)
*T2*	9 (47.3%)
*T3*	3 (15.7%)
Neoadjuvant therapy	9 (47.3%)
Type of anastomosis	
*Mechanical double stapling*	16 (84.2%)
*Coloanal hand-sewn*	3 (15.7%)

## Data Availability

The data presented in this study are available on request from the corresponding author.
